# Study protocol: associations between dietary patterns, cognitive function and metabolic syndrome in older adults – a cross-sectional study

**DOI:** 10.1186/s12889-019-6900-4

**Published:** 2019-05-10

**Authors:** Karen D. Mumme, Pamela R. von Hurst, Cathryn A. Conlon, Beatrix Jones, Crystal F. Haskell-Ramsay, Welma Stonehouse, Anne-Louise M. Heath, Jane Coad, Kathryn L. Beck

**Affiliations:** 1 College of Health, Massey University, Private Bag 102904, Auckland, 0745 New Zealand; 2 Department of Statistics, University of Auckland, Private Bag 92019, Auckland, 1142 New Zealand; 3 Department of Psychology, Northumbria University, Newcastle-upon-Tyne, NE1 8ST UK; 4 Health and Biosecurity Business Unit, Commonwealth Scientific Industrial Research Organisation, PO Box 11060, Adelaide, SA 5001 Australia; 5 Department of Human Nutrition, University of Otago, PO Box 56, Dunedin, 9054 New Zealand; 6 College of Sciences, Massey University, Private Bag 11222, Palmerston North, 4442 New Zealand

**Keywords:** Dietary patterns, Cognitive decline, Cognition, Metabolic syndrome, Older adults, Principal component analysis, New Zealand

## Abstract

**Background:**

Loss of cognitive function is a significant issue as the world’s population ages. Preserving cognitive function maintains independence in older adults bringing major societal and financial benefits. Lifestyle factors such as diet are modifiable risk factors, which may help preserve cognitive function.

Most nutrition research aimed at preserving cognitive function and metabolic health has focussed on individual nutrients and foods, not allowing for food combinations and interactions. A dietary pattern approach considers the entire diet including its complexity. Previous research investigating dietary patterns and cognitive function has not always considered relevant covariates such as physical activity and the Apolipoprotein E genotype, which are known to have associations with cognitive function.

The aim of the REACH (Researching Eating, Activity and Cognitive Health) study is to investigate associations between dietary patterns, cognitive function and metabolic syndrome, accounting for a range of covariates.

**Methods:**

This cross-sectional study design will recruit older, community-living adults (65–74 years) from Auckland, New Zealand. Dietary data will be collected via a 109-item food frequency questionnaire validated using a 4-day food record. Cognitive function will be assessed using the Montreal Cognitive Assessment (paper based) and the Computerised Mental Performance Assessment System (COMPASS) - a testing suite covering six domains. Additional data will include genetic (Apolipoprotein E ε4) and biochemical markers (fasting glucose, HbA1c, lipids profile), anthropometric measurements (weight, height, waist and hip circumference, body composition using dual X-ray absorptiometry), blood pressure, physical activity (International Physical Activity Questionnaire – short form) and health and demographics (questionnaire).

Dietary patterns will be derived by principal component analysis. Associations between cognitive function and dietary patterns will be examined using multiple regression analysis. Covariates and interaction factors will include age, education, socio-economic status, physical activity, Apolipoprotein E ε4 genotype, family history of dementia or cognitive impairment, and lifestyle factors. Differences between participants with and without metabolic syndrome will also be examined.

**Discussion:**

This study will bring new knowledge regarding associations between dietary patterns and cognitive function and metabolic health in older adults living in New Zealand. This is important for developing nutrition related recommendations to help older adults maintain cognitive function.

## Background

The global population aged over 60 years has doubled in the last 30 years and is expected to double again by 2050 [1]. Independence and quality of life are valued by older adults [2] but these attributes are threatened by poor health in later years [3]. Delaying entry of older adults into aged care residential facilities brings substantial financial benefits to individuals and governments [4]. A key predictor for admission into residential care is impaired cognitive function or dementia [5]. Loss of cognitive function takes away independence, a healthy fulfilling life in final years and can significantly affect family members [6]. Hence, there is a case for preserving cognitive function for as long as possible.

Age-related cognitive decline, mild cognitive impairment and dementia are progressive with limited pharmacological and non-pharmacological treatments available [7]. Without a cure, researchers should strive to discover ways to prevent or slow cognitive decline and the onset of dementia through lifestyle and dietary changes [6].

Age and genetic factors are the most significant risk factors for developing dementia [6]. One specific genetic factor, Apolipoprotein E ε4 (ApoE ε4) not only increases the risk for developing Alzheimer’s Disease [8] and vascular dementia [9] but also puts healthy individuals at risk of a faster cognitive decline as they age [10]. Age and genotype cannot be altered but there are some modifiable risk factors [11] which may slow cognitive decline.

Modifiable risk factors include physical inactivity, smoking, hypertension, obesity, type 2 diabetes mellitus and low educational attainment [11]. Three of these risk factors (hypertension, obesity and diabetes) are components of metabolic syndrome, which further suggests the presence of a “metabolic-cognitive syndrome” via their similar underlying mechanisms through vascular changes [12]. The relevance of lifestyle and diet in cognitive decline and metabolic syndrome is well-recognised [13] and lifestyle and diet is a starting point in the prevention, delay or reduction of the impact of cognitive decline.

Foods and nutrients that may protect against the loss of cognitive function have been identified. These include fruits and vegetables [14–16], fish [15, 16], mono- and poly-unsaturated fats [15, 16] and antioxidants [17, 18]. In contrast, nutrients such as refined carbohydrates and saturated fat may impair cognitive function [15, 19].

Studies of food groups and macro- and micro-nutrients make a valued contribution to nutritional science. However, people consume a diet consisting of many foods, and single nutrient or food group studies may have outcomes confounded by the overall complexity of the diet. The food combinations within a diet can act synergistically, for example altering the bioavailability of nutrients within the body. These changes are not considered in studies of individual food groups or nutrients alone [20]. The study of the combinations and complexities of dietary intake is a more recent development and dietary pattern analysis acknowledges the complexities of food combinations and their associated biochemical interactions as important [21].

Dietary patterns are derived through two processes using dietary data. The first process uses theoretically derived (*a priori*) dietary patterns based on current nutritional knowledge. A dietary index is created, ranking study participants’ adherence to dietary guidelines (e.g. US dietary guidelines) or a dietary pattern (e.g. the Mediterranean diet index) [20]. Several studies show better cognitive function in older adults if their normal dietary intake follow specific features of a diet, for example the Mediterranean diet with its high intake of vegetables, fruits, fish, nuts, cereals and olive oil [16] or the Dietary Approach to Stop Hypertension (DASH) diet, high in vegetables and dairy with a low consumption of saturated fats and sodium [15]. Additionally, the Mediterranean diet, DASH and a Nordic diet, based on Nordic nutrition recommendations, may also have benefits with regards to metabolic syndrome [22].

The second process is an empirically derived (*a posteriori*) dietary pattern. The statistical method of principal component analysis allows measurements on many dietary components to be reduced to measures on a few different dietary patterns [20]. This method enables a better understanding of eating behaviours and a unique view of dietary intake [23] within a population setting. With regards to the older adult, positive associations between empirically derived dietary patterns and cognitive function have been recognised in ‘healthy/prudent’ [24–26] or ‘carotenoid-rich’ [27] dietary patterns in primarily European or North American populations and ‘plant food and fish’ [28], ‘vegetables-fruits’ [29] and ‘snack-drinks-milk products’ [29] dietary patterns in Asian populations. Conversely, negative associations have been noted in cognitive function where a ‘Western’ [25] dietary pattern was consumed. In some instances, no associations were found between cognitive function and ‘traditional’ [26], ‘meat-fish [29], ‘health aware’ [30] or ‘sweet foods’ [30] dietary patterns.

Associations can be attenuated when important covariates are included in the statistical analysis suggesting that the covariate has an effect on the outcome. For example, negative associations between cognitive function and ‘high red meat’ [31], ‘high butter’ [31], ‘processed food’ [32] and ‘traditional’ [30] dietary patterns were weakened when the ApoE ε4 genotype [31], education [32] or childhood IQ [30] were considered in the analysis. Likewise, a positive association was weakened in a ‘whole food’ and ‘Mediterranean style’ dietary pattern with education [32] or childhood IQ [30] as covariates. Consideration of covariates is critical; without it some studies may not be as robust and lead to misinformed conclusions. One of these covariates is the ApoE ε4 genotype. This genotype has been considered in recent research. Analyses show associations are still apparent between dietary patterns and cognitive function when ApoE ε4 is adjusted for [33–35], though in older studies this covariate was disregarded [24–26, 29, 32, 36].

A ‘dietary pattern’ is population specific and dietary patterns have been studied in the New Zealand context [37–42]. However, no studies have investigated dietary patterns exclusively in an older New Zealand population. Therefore, the objectives of the REACH (Researching Eating, Activity and Cognitive Health) study are to investigate associations between dietary patterns and cognitive function in older adults within the New Zealand population. A secondary objective is to investigate associations between dietary patterns and metabolic syndrome.

## Methods

### Study design and participants

The participants in this cross-sectional study will be men and women aged 65–74 years, living independently in Auckland, New Zealand and proficient in English. Participants will be excluded if they are colour blind (due to the computerised cognitive testing requiring colour recognition); have a diagnosis of dementia or any of the following conditions which may impair cognitive function: stroke, traumatic head or brain injury, a neurological or psychiatric condition; or if they are taking medication which may influence their cognitive function. Another exclusion criterion is any event in the last 2 years which had a substantial impact on dietary intake and cognitive function, for example, death or illness of a family member.

A sample size of 350 participants is required to see a medium size effect (Pearson correlation 0.3) with 80% power for the main outcome of a linear association between the cognitive scales and dietary patterns. This sample size is similar to other studies investigating cognitive function and dietary patterns [36]. Data will be collected from 360 participants to allow for missing or incomplete data.

Recruitment and data collection will commence in 2018 and is expected to take 12 months. Participants will be required to attend the Human Nutrition Research Unit, Massey University, Auckland, New Zealand on one occasion. Informed consent forms will be completed at the research facility prior to data collection. The research day involves collecting health and demographic data, blood pressure, anthropometric and physical activity data as per Table 1. A fasted blood sample will be taken prior to a standardised breakfast. Two cognitive assessments will be completed after breakfast. An online food frequency questionnaire (FFQ) and a food diary information video complete the session.

**Table 1:** Outcome measures and testing methods for data collection

Variables	Methods
Questionnaires	
Health and demographics	Written questionnaire developed by the researchers – questions regarding: socio-demographic, health, lifestyle and dietary factors.
Physical Activity	International Physical Activity Questionnaire - short form [43]
Anthropometry	
Height, weight, waist and hip circumference	ISAK^b^ anthropometry methods [44] – stadiometer, Tanita Electronic Scales, Lufkin W600 PM flexible steel tape
Muscle mass and fat mass	Dual-emission X-ray absorptiometry, Hologic, Discovery QDR series
Blood Analysis	
Fasting blood glucose	HemoCue Glucose 201RT
HbA1c	Cobas b 101 system [45]
Lipid Profile (total cholesterol, triglycerides, HDL-C, LDL-C^a^)	Cobas b 101 system [45]
Apolipoprotein E ε4	Polymerase chain reaction amplification and direct nucleotide sequence analysis
Clinical	
Blood pressure	Digital Automatic Blood Pressure Monitor, Omron HEM-907
Dietary intake	
Food Frequency Questionnaire	Via Survey Monkey – adapted from Beck et al. [46]
Estimated 4-day food diary	Paper form
Cognitive tests	
Global cognitive function	Montreal Cognitive Assessment (MoCA) [47]
Multiple cognitive domains	Computerised Mental Performance Assessment System (COMPASS-Northumbria University, Newcastle upon Tyne, UK) refer Table 2

^a^calculated

^b^International Society for the Advancement of Kinanthropometry

Funding is provided by the Health Research Council of New Zealand, Grant 17/566. Ethical approval has been granted by Massey University Human Ethics Committee: Southern A, Application 17/69.

### Recruitment and screening

Participants will be recruited throughout the wider Auckland region via several channels including: the Human Nutrition Research Unit, Massey University participant database; media, including radio interviews and press releases from Massey University; posters and flyers at local libraries, community centres, recreation centres, sports and hobby clubs, Citizens Advice Bureaus, retirement villages and second hand shops; inclusion in relevant newsletters, e.g. Age Concern New Zealand; and online promotion on appropriate social media pages e.g. GrownUps, Office for Seniors Facebook page. The REACH study will have a website where potential participants will be directed for further information and to register interest [48]. Only one person per household will be eligible to participate in the study. Participants expressing an interest in the study will be provided with an information sheet and undergo a screening interview [48], via telephone, to ensure inclusion criteria are met.

### Health, demographic, and physical activity data

Health, demographics, lifestyle and physical activity information will be collected through written questionnaires in person (Table 1). Data quality will be ensured by checking questionnaires for completeness and sensibility. If further clarification is required, it will be done immediately or within a few days by phone or email. Socio-demographic information includes age, gender, ethnicity, marital status, education, household income, work history, living situation and food security. Health information will include past and current disease (acute and chronic), medication, family history of dementia or cognitive impairment, vision, hearing, dental health and mobility issues. Lifestyle information will include smoking history, alcohol intake, substance abuse, supplement use and changes in physical activity. Dietary information will include changes in diet over past 10 years and possible factors behind these changes e.g., dentition, health concerns, disposable income, appetite. An Index of Multiple Deprivation will be calculated from the residential address of participants [49].

### Anthropometric data and blood pressure

Anthropometry, including body fat percentage, BMI, hip and waist circumference, and blood pressure will be measured as in Table 1.

Blood pressure will be measured twice. Participants will rest quietly (seated) for 5 min before the first measurement is taken. There will be a 1-min rest period between measurements. If either systolic or diastolic measurements differ by more than 5 mmHg between measurements, a third measurement will be taken.

### Blood sampling, processing, analysis and genotyping

For consistency, all participants will be fasted (except water) from 2200 h the night prior to their visit. Between 0700 and 0900 at the research facility, a qualified phlebotomist will draw fasted blood samples in the following order: 10 ml BD Vacutainer® Plus (cat 367895), 10 ml BD Vacutainer® K2EDTA (cat 367525) and 6 ml BD Vacutainer® K2EDTA (cat 367873). The 10 ml BD Vacutainer® Plus will be maintained at an ambient temperature for 30 min to allow clotting, then will be centrifuged with the 10 ml BD Vacutainer® K2EDTA (Heraeus Labofuge 400R) for 15 min at 1547 g-force (3500 rpm) at 4 °C. The resulting serum, from the 10 ml BD Vacutainer® Plus, will be aliquoted into four Eppendorf tubes which will be stored for further measures. The resulting plasma, from the 10 ml BD Vacutainer K2EDTA, will be aliquoted into six Eppendorf tubes and stored for further research. The 6 ml EDTA vacutainer will be placed on ice and tested for blood glucose, HbA1c and a lipid profile using point-of-care equipment (Table 1). The remaining blood (~ 5 ml) will be stored for ApoE ε4 analysis (Table 1) by an accredited laboratory, and two Eppendorf tubes (500 μl) will be kept for backup. All samples will be stored at -80 °C.

### Assessment of dietary intake

The study spans a 10-month period from autumn to summer. Dietary data will be collected using two methods. First, a self-administered 109-item FFQ will collect data, covering the previous month, via an online survey on two occasions: at the research facility (FFQ1) and 1 month later (at home) (FFQ2) to assess reproducibility. The FFQ is adapted from a validated New Zealand FFQ aimed at assessing iron related dietary patterns in young women [46]. Changes included the addition of serving sizes; combining some food groups to shorten the questionnaire; the addition of foods not included in the initial nutrient specific FFQ e.g. confectionary; as well as extra questions for added clarification e.g. type of milk or oils used. The FFQ was further cross-checked with the New Zealand Women’s Food Frequency Questionnaire [50, 51] to ensure all relevant food groups were included. Ten individuals in the study age range pre-tested the FFQ for understanding and readability. The completed FFQ will be entered into Foodworks 9 (Xyris Software, 2017), which uses the New Zealand FOODfiles^(^™^)^ 2016 [52] food composition database. After data entry and inspection, any necessary adjustments will be made e.g. Goldberg Cut-off for energy intake [53].

Second, an estimated 4-day food diary will be collected from a subset of participants to validate the modified FFQ for food and dietary patterns. Participants will complete the 4-day food diary within 1 month of the study visit. The 4-day food diary covers four consecutive days including at least one weekend day. Prior to completing the 4-day food diary, participants will view an instructional video which explains the need to record all foods and beverages consumed including type, brands, and cooking methods. Participants will be taught how to estimate quantities using pictures [54], household measures, and measuring scales. The 4-day food diary will be processed by four trained nutritionists using Foodworks 9 (Xyris Software, 2017). A register of common food items will be kept ensuring consistency in data entry among the four nutritionists. Additionally, every tenth food record entered will be audited for accuracy and consistency.

### Cognitive assessment

Cognitive testing will be carried out after a standardised breakfast to minimise any effects food may have on cognition. The cognitive testing will be undertaken in two parts. First, a trained examiner will administer the Montreal Cognitive Assessment (MoCA) on a one to one basis. MoCA is a clinically available and validated [47] tool. It takes 10 min, assesses global cognitive function, short-term memory, visuospatial and executive function, attention, language and orientation, concentration and working memory [47]. MoCA will be used to provide comparisons with other studies, and as a descriptor.

The second part will be the Computerised Mental Performance Assessment System (COMPASS-Northumbria University, Newcastle upon Tyne, UK). This software platform presents tasks on desktop computers using a variety of methods to collect responses: mouse and cursor, a four-button coloured response pad, and pen and paper for word recall. This broad battery of tests assesses all cognitive domains (Table 2) and is sensitive to normal age-related effects and dietary factors [55, 56].

**Table 2:** The COMPASS^a^ battery of assessments

Cognitive Domain	Definition	Test
Mood	Measurement of subjective feelings	Bond and Lader Mood Scales
Attention and vigilance	Attention - ability to concentrate on selected aspects of the environment while ignoring other stimuli	Simple reaction time
		Choice reaction time
		Digit vigilance task
	Vigilance - ability to maintain attention and alertness over time	
Executive function	Co-ordination of cognitive responses – sub-serves planning, initiating and inhibiting actions, cognitive flexibility, abstract thinking and rule acquisition	Stroop test
Episodic memory	Ability to retain memories that can be consciously recorded e.g. facts, items, events, faces	Immediate and delayed word recall
		Delayed word recognition
		Delayed picture recognition
Working memory	Ability to hold information in mind while carrying out more complex cognitive processes	Corsi blocks
Location learning	Assesses visuo-spatial memory	Computerised location learning
		Computerised location recall

^a^Computerised Mental Performance Assessment System (COMPASS-Northumbria University, Newcastle upon Tyne, UK)

The reaction time for correct responses will be assessed and participants will be assigned a composite score for global cognitive function and for each cognitive domain.

Cognitive testing will be undertaken in a cognitive suite controlled for environmental factors e.g. noise and temperature. The tests will be taken at a similar time of day and participants will be instructed to avoid undue stress, alcohol, recreational drugs and physical activity that is not routine prior to their appointment. Testing will take approximately 1 h. The first 15 min will be training. The investigator will verbally describe the tasks, the format of the testing, and use of the response pad, and will answer any questions. The practice tests will be shorter and easier versions of the actual test. Participants will have a 5-min break before actual assessment is undertaken. The investigator will ensure any new computer users are comfortable with using a mouse before testing starts.

### Metabolic syndrome

To determine the incidence of metabolic syndrome the criteria recommended by the American Heart Association/National Health, Lung and Blood Institute Scientific Statement will be followed [57]. Metabolic syndrome will be considered to exist where three of the following five criteria are met or medication is used to treat: waist circumference ≥ 88 cm for women and ≥ 102 cm for men; a triglyceride level of ≥1.7 mmol/L; HDL cholesterol level of < 1.03 mmol/L in men or < 1.3 mmol/L in women; blood pressure ≥ 130/85 mmHg; fasting blood glucose ≥5.6 mmol/L.

### Provision of results to participants

Feedback to participants after assessment will include anthropometric measurements (height, weight, BMI, waist and hip circumference, body fat %), blood pressure and blood results (HbA1c, fasting blood glucose, lipid profile). A registered (NZ Medical Council) general practitioner will review all biochemical results prior to communicating them to participants. On completion of the study, participants will receive a report summarising the main findings of the REACH study.

### Statistical analysis

Statistical analysis will be performed using R [58]. Participant data will be described using mean (95% confidence intervals) for normally distributed data, median (25, 75 percentile) for non-normally distributed data, or frequency summary statistics for categorical data. The Shapiro-Wilk test and normality plot will evaluate the normality of distributions.

Dietary patterns will be identified using rotated principal component analysis, a statistical technique to reduce data and produce patterns based on the correlations between food groups [23]. The FFQ food group items will be further collapsed based on other studies investigating dietary patterns [40, 41]. Three separate analyses will be performed: FFQ1 to determine dietary patterns, the 4-day food diary to assess validity of the dietary patterns identified in FFQ1, and FFQ2 vs FFQ1 to assess reproducibility. Orthogonal varimax rotation will be used to facilitate interpretability of components. The number of components retained will be based on the scree plot, eigenvalue (> 1), and interpretability of the dietary pattern. The Kaiser-Meyer-Olkin measure of sampling adequacy and Bartlett’s Test *P* values (to determine the presence of relationships between variables in the factor analysis) will be examined [59]. Labelling of dietary patterns will be based on the interpretation of foods with high factor loadings for each dietary pattern [60].

Multivariate multiple regression analysis [61] will be used to determine the association between dietary patterns and various domains of cognitive function (dependent variable) while considering confounding factors. Possible confounding and interaction factors include: age, gender, ethnicity, education, English as a second language, presence of chronic disease (including metabolic syndrome), socio-economic status, physical activity, body mass index, ApoE ε4 genotype, family history of dementia, smoking, alcohol intake and past dietary intake.

Further analysis will compare dietary patterns of participants with and without metabolic syndrome using multiple logistic regression analysis.

## Discussion

Age-related cognitive decline is a continuum of natural cognitive changes that may progress into mild cognitive impairment or dementia. Rates of decline differ within a population. Delaying the onset of cognitive decline is the optimal strategy but where there is an earlier on-set the ability to slow the decline is important. Delaying or slowing the decline may be abetted by modifiable lifestyle factors e.g. diet, physical activity. Therefore, an in-depth understanding of the diet (beyond an isolated food and nutrient approach) of older adults is warranted. The REACH study will broaden the current knowledge on dietary patterns and their associations with cognitive function and metabolic health in older adults in a New Zealand context.

Despite the cross-sectional design having limitations e.g. causality cannot be inferred, the study design has several strengths. The study recognises dietary patterns as a measure of the diet and acknowledges the whole diet is greater than the sum of its parts. Extra strengths come from the collection of data on the ApoE ε4 allele, physical activity levels and other known covariates to ensure that any association found is likely to be due to the dietary patterns identified. Additionally, the COMPASS battery of cognitive tests is administered on the computer minimising potential bias associated with administration of these tests by a researcher. The scoring of cognition and the assessment of dietary patterns will be done independently to minimise bias. While there is always bias associated with the type of participant attracted to such studies, the bias will be minimised by using a range of recruiting methods across the wider Auckland region to ensure a range of participant demographics are captured. Furthermore, the size of the study population ensures there is adequate power to detect a meaningful result.

The REACH study is the first to examine dietary pattern associations in older New Zealand adults. This first step paves the way for developing recommendations for New Zealanders to maintain cognitive function, metabolic health and ultimately quality of life as they age. Additionally, it will provide a valuable base for hypothesis generation for future longitudinal studies and / or randomised controlled trials.

## Abbreviations

ApoE ε4: Apolipoprotein E ε4
COMPASS: Computerised Mental Performance Assessment System
DASH: Dietary Approach to Stop Hypertension
FFQ: Food Frequency Questionnaire
FFQ1: First Food Frequency Questionnaire
FFQ2: Second Food Frequency Questionnaire
ISAK: International Society for the Advancement of Kinanthropometry
MoCA: Montreal Cognitive Assessment
